# Implementation of Customized Homogenizing Devices in Radiotherapy for the Treatment of Non-Melanoma Skin Cancer: Three Case Reports

**DOI:** 10.3390/reports8030165

**Published:** 2025-09-01

**Authors:** Rosa Marene Hernández Martínez, Juan Carlos Torres Velasco, Alejandro Chagoya González, Carlos Alberto Castro-Fuentes, Kuautzin Alfonso Hernández

**Affiliations:** 1 Departamento de Prótesis Maxilofacial, Hospital Regional de Alta Especialidad de Ixtapaluca, Servicios de Salud del Instituto Mexicano de Seguro Social Para el Bienestar (IMSS-BIENESTAR), Carretera Federal México-Puebla Km 34.5, Ixtapaluca 56530, CP, Mexico; pmf.marene.hernandez@gmail.com; 2 Hospital de Especialidades con Oncología de Gómez Palacio, De Las Violetas 1152, Rinconadas Bugambilias, Gómez Palacio 35010, CP, Mexico; carlostove@gmail.com; 3 Departamento de Radioncología, Hospital Regional de Alta Especialidad de Ixtapaluca, Servicios de Salud del Instituto Mexicano de Seguro Social Para el Bienestar (IMSS-BIENESTAR), Carretera Federal México-Puebla Km 34.5, Ixtapaluca 56530, CP, Mexico; dr.alejandrochagoya@gmail.com; 4 Unidad de Investigación, Hospital Regional de Alta Especialidad de Ixtapaluca, Servicios de Salud del Instituto Mexicano de Seguro Social Para el Bienestar (IMSS-BIENESTAR), Carretera Federal México-Puebla Km 34.5, Ixtapaluca 56530, CP, Mexico

**Keywords:** radiotherapy, homogeneity, bolus, dosimetry, non-melanoma skin cancer, case report

## Abstract

**Background and Clinical Significance**: In most cases, the success of radiotherapy in the treatment for skin cancer is limited, particularly due to the irregularities of the neoplasm’s surfaces or even tissue discontinuity. Based on a comprehensive clinical assessment, the therapeutic approach for radiotherapy was established for the patients included in this study. Wax-paraffin (50:50) devices were custom-designed for radiotherapy treatment, confirming adequate homogeneity and conformity indices for doses of 55–66 Gy, and chemotherapy when necessary. Toxicity and treatment response were also assessed; **Cases Presentation**: For patient 1, two lesions located on the right nasolabial fold and right thigh were treated with radiation, and a 1 cm thick wax-paraffin surface bolus was designed, allowing for improved dose distribution and favorable local response. For patient 2, in addition to the thick wax-paraffin homogenizer, lead eye protectors were designed due to the location of the tumor, with the aim of protecting organs at risk. The treatment in this patient resulted in effective local response. Finally, for patient 3, with a lesion in the supraclavicular region extending to the left shoulder due to acantholytic squamous cell carcinoma with secondary carcinomatous lymphangitis, 1 cm thick wax-paraffin surface homogenizers were used; **Conclusions**: Due to the characteristics of the customized homogenizers, tumor lesion remission was successfully achieved in all three patients, highlighting both the advantages of these devices and their efficacy in dose distribution and local response in radiotherapy treatment of non-melanoma skin carcinoma.

## 1. Introduction and Clinical Significance

Skin cancer is one of the most common types of neoplasia worldwide, with an incidence of 1,234,533 new cases in 2022 [1]. Particularly, non-melanoma skin cancer (NMSC) is the most common type of neoplasia, with an increasing incidence attributed to factors such as prolonged unprotected sun exposure, aging population, and use of tanning beds, and an increase in immunosuppression at younger ages [2].

Regarding NMSC, the most common types of skin cancer are basal cell carcinoma (BCC) and cutaneous squamous cell carcinoma (cSCC), representing more than 95% of cases [2]. These cancers typically develop in areas such as the face and hands, and recent studies have identified genetic and environmental factors as important triggers [2,3], underscoring the need to improve clinical registries and preventive strategies, especially in developing countries like Mexico [4].

Mohs surgery remains the gold standard for localized tumors due to its high cure rates, while superficial radiotherapy is successfully used for low-risk carcinomas with cure rates of 90–95% [5,6]. The latter uses ionizing radiation to deliver a radiation dose to the tumor sufficient to destroy it, while sparing the surrounding healthy tissue as much as possible [6,7].

In cases of facial tumor diagnoses, an evaluation by a radiation oncologist and maxillofacial prosthetist is necessary to determine whether the creation of accessories is required as part of the radiation treatment plan [2]. These devices are designed from an impression of the affected area, resulting in the generation of customized models to provide greater efficacy in radiation therapy in its various forms [8,9,10].

Homogenizing molds are prosthetic devices designed to fill in the surface irregularities of neoplasms or to replace lost tissue continuity after surgical tumor resection. Their purpose is to create a uniform plane that allows for homogeneous distribution of the radiation beam throughout the treatment field, adapting to the needs of each patient [11,12,13]. However, few studies analyze the advantages of their use and their impact on conformity and homogeneity rates in radiotherapy, primarily in developing countries such as Mexico.

Therefore, the present study evaluates the benefits and the dosimetric indexes of conformity and homogeneity associated with customized homogenizing devices for the treatment of non-melanoma skin cancer in three patients managed at a tertiary-level hospital in Mexico.

## 2. Cases Presentation

Table 1 summarizes the clinical data, including histology, location, acute and chronic toxicity, dose, clinical response, follow-up, and conformity and homogeneity indexes of non-surgical cases according to the guidelines NCCN [14,15] successfully treated with radiotherapy and homogenizers. Regarding histology, the three cases (discussed below) presented solid infiltrating basal cell carcinoma, classic, and squamous cell carcinoma. The carcinoma was primarily located on the upper trunk. In all cases, dermatitis was the acute presentation, while hypopigmentation was chronic. Only one case was treated with chemotherapy (Patient 3). A complete clinical response was achieved in all cases after an average follow-up of 2 years. The average conformity and homogeneity indexes were 0.417618 and 0.112798, respectively.

### 2.1. Case 1

**Figure 1:**
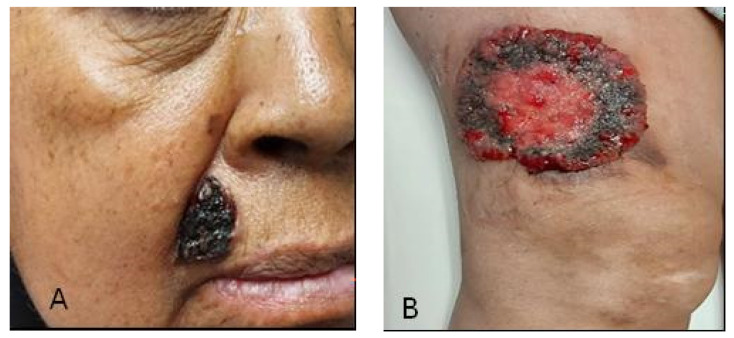
(**A**) Basal cell carcinoma of the nasolabial fold; (**B**) Basal cell carcinoma of the right thigh.

A 58-year-old female patient with a maternal history of cancer (gastric cancer) and a personal history of childhood poliomyelitis with motor sequelae. Upon evaluation, a lesion located in the right nasolabial fold of 2.5 × 3 cm (Figure 1A) and a 10 cm diameter lesion on the right thigh (Figure 1B) were identified. A CT scan was performed to evaluate initial extension; however, no locoregional or distant metastatic activity was reported. According to the histopathological report, the diagnosis was infiltrating solid basal cell carcinoma with an adenoid component [14,15]. Therefore, radiotherapy management was performed with a dose of 55 Gy in 20 fractions with electrons.

A customized homogenizer of wax-paraffin (50:50) with a thickness of 1 cm was designed to provide adequate radiotherapy treatment. In this paper, a homogenizer in radiation physics is defined as a tissue-equivalent material that maximizes or reduces the radiation dose to an irradiated area. This material will be defined by the volumes established under the guidelines of the International. Commission on Radiation Units and Measurements (ICRU 24) [16].

The prescribed dose for the Planning Target Volume (PTV) exceeded 95% coverage, and the dose received by the at-risk organs was within the range established by the Quantitative Analyses of Normal Tissue Effects in the Clinic (QUANTEC) [17]. This was confirmed by Dose-Volume Histogram (DVH) analysis.

To evaluate the efficacy of oncological radiotherapy treatment using customized homogenizers, it was necessary to evaluate the conformity index considering the conformity index value ranges according to Yoon M et al. [18].

The homogeneity index is a measure of dose differences within the tumor target depending on the maximum dose, defined in the equation. Depending on the literature reviewed, 0.7 is cited as the optimal value, while others cite it as 1. Therefore, in the present study, whenever the HI was ≤2, the treatment was considered to comply with the protocol.

Once conformity and homogeneity indexes were met, radiotherapy dose assessment was performed for each patient using the customized homogenizer (wax-paraffin with Hounsfield Units (HU) like fatty tissue −100 HU).

**Figure 2:**
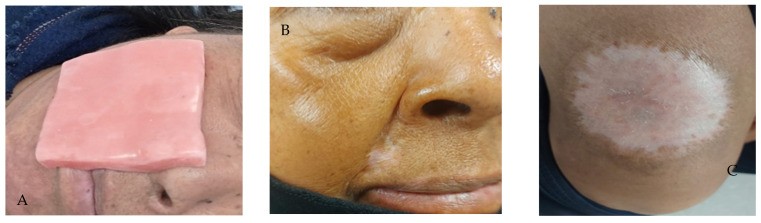
(**A**) Wax-paraffin based surface homogenizer customized; (**B**) Total clinical response with signs of hypopigmentation of the asogenous sulcus; (**C**) Total clinical response with evidence of hypopigmentation in the thigh.

A 1 cm thick wax-paraffin based surface homogenizer customized for both regions (Figure 2A), adding 2 cm to the tumor margin, was designed; in addition, 6 MeV (Megaelectronvolt) energy was used, with a 15 cm cone, and a linear accelerator with cerrobend shieldings. Figure 2B,C show the clinical response to radiation treatment with the use of homogenizers, where a complete clinical response is evident.

### 2.2. Case 2

**Figure 3:**
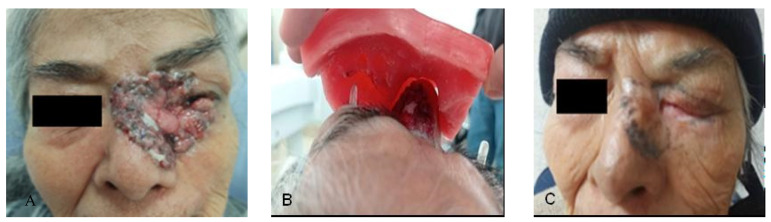
(**A**) Basal cell carcinoma of the inner canthus of the left eye; (**B**) Placement and adjustment of wax and paraffin homogenizers on the stem and volume of the eye shields; (**C**) Total clinical response with skin hypopigmentation and hyperpigmentation.

An 88-year-old female patient with no relevant family history presented with a nodular lesion in the left nasal region involving the inner canthus of the left eye (Figure 3A), as well as the ipsilateral upper and lower eyelid, measuring approximately 6 × 5 cm. A CT scan was performed to evaluate initial extension; however, no locoregional or distant metastatic activity was reported. Histopathological assessment of the lesion revealed classic basal cell carcinoma. The treatment was carried out with 55 Gy in 20 fractions with photons and placement of custom lead eye shields, in addition to a 1 cm thick wax-paraffin (50:50) homogenizer, taking into consideration the conformity and homogeneity indexes for its design (Figure 3B).

Our patient was observed with grade 1 radiodermatitis skin toxicity, grade 1 lower eyelid retraction, and grade 1 dry eye with decreased eyelid closure according to CTCAE [19]. The assessment at 5 months after treatment remained without evidence of locoregional tumor activity, skin with grade 1 hyperpigmentation and grade 1 hypopigmentation, maintained grade 1 eyelid retraction and grade 1 dry eye according to CTCAE v5.0 [19]. Currently in follow-up and surveillance, with no evidence of local tumor recurrence (Figure 3C).

### 2.3. Case 3

**Figure 4:**
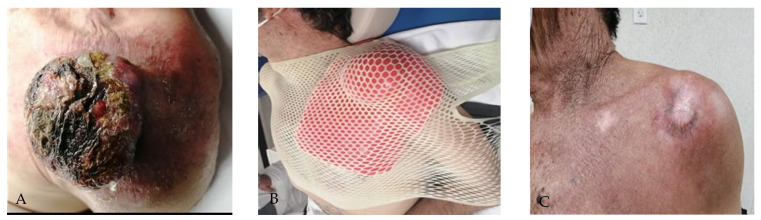
(**A**) Squamous cell carcinoma on the left shoulder; (**B**) Customized paraffin wax homogenizer with thermoplastic mesh fixation; (**C**) Complete clinical response with skin hypopigmentation.

A 78-year-old male patient with no family history presented with a supraclavicular lesion extending to the left shoulder, with an 11 cm tumor (Figure 4A). A CT scan was performed to evaluate initial extension; however, no locoregional or distant metastatic activity was reported. The histopathological report confirmed acantholytic squamous cell carcinoma with carcinomatous lymphangitis secondary to squamous cell carcinoma.

The patient received 4 cycles of CPPD chemotherapy and concomitant radiotherapy, with a dose of 66 Gy in 33 fractions, delivered in two phases. A customized 1 cm thick wax-paraffin (50:50) surface homogenizer was used for the first phase, followed by a second customized 1 cm thick wax-paraffin (50:50) homogenizer as tumor volume decreased (Figure 4B).

Initially, this patient presented with grade 1 skin toxicity based on CTCAE v5.0 criteria [19]. The patient had a complete clinical and imaging response, followed by chronic toxicity with grade 1 hypopigmentation, and remained disease-free for 3 months (Figure 4C). However, at 9 months, pulmonary and locoregional progression was evident. Therefore, palliative chemotherapy was initiated. The patient died 13 months after the end of treatment.

## 3. Discussion

In radiotherapy, beam dosimetry is performed on a phantom, requiring a flat surface perpendicular to the central beam at the time of treatment. However, due to irregularities in the area to be irradiated, not all beams travel the same distance in the air on one side of the field. Furthermore, tissue thickness can limit adequate dose delivery [20]. Therefore, it is essential to perform a correction, such as using compensating homogenizers with absorbing and scattering properties that simulate those of soft tissue and are therefore suitable as a filling material between the end of a cone and the skin where the air space occurs [20].

Currently, it is necessary that within the clinical and comprehensive approach to the patient diagnosed with a facial tumor, the radiation oncologist, medical physicist, and maxillofacial prosthetist jointly evaluate the intensity-modulated radiotherapy treatment plan for head and neck cancers, due to the dosimetric uncertainty in the surface regions to be irradiated, due to their irregularity [21]. In this context, previous studies such as those by Chung et al. [21], have evaluated the surface dose and the accumulation region for intensity-modulated radiotherapy in head and neck cancer. The authors used pieces of radiochromic film for in silico dose measurement and thus determined the dosimetric discrepancies in the surface and accumulation region between the prediction of the Treatment Planning System and the experimental measurement using the radiochromic film. The authors determined two targets, one shallow (0.5 cm depth) and one deep (6 cm depth), at a dose of 54 Gy. Generally, good agreement was found with the measurements. However, significant discrepancies were identified from the surface to approximately 0.2 cm depth in both cases.

Additionally, Qi et al. [22] verified the surface dose for head and neck treatments using intensity-modulated radiation techniques. Using a metal-oxide-semiconductor field-effect transistor (MOSFET) detector, in vivo cutaneous dosimetry was determined for the treatment of nasopharyngeal carcinoma with modulated radiation therapy in a phantom and in patients. The results showed that the MOSFET detector, encapsulated in a thin water-protective film, exhibits minimal and reproducible intrinsic water accumulation, which is recommended for cutaneous dosimetry.

However, dosimetric uncertainty in radiotherapy due to the customized device used, as well as the development of impractical and expensive technologies for routine treatment in developing countries such as Mexico, are limitations.

**Figure 5:**
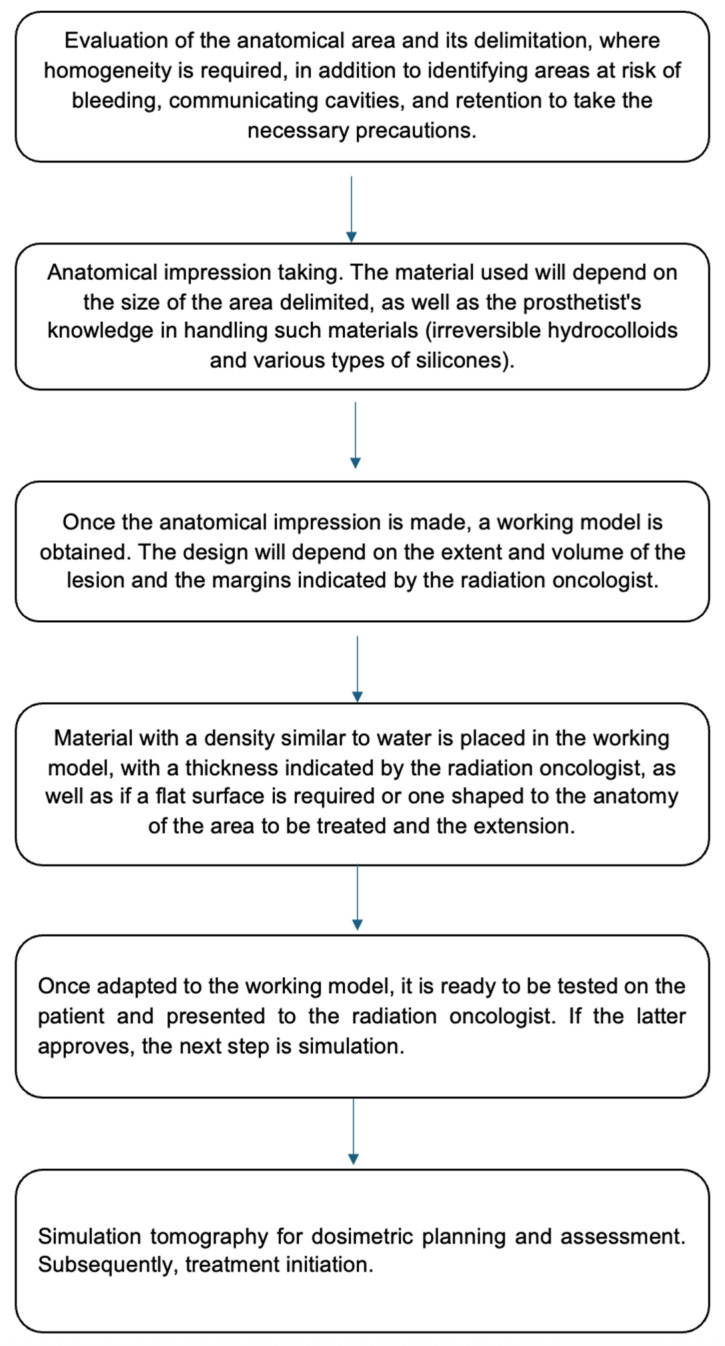
Flowchart of the approach process, design of personalized homogenizers, and radiotherapy treatment in patients with skin cancer.

Endarko et al. [23] reported that silicones, natural waxes, and paraffin, as well as some types of plasticine, have adequate densities comparable to those of breast, fat, and muscle tissue. Recently, Boopathi et al. [8] fabricated and evaluated the dosimetric characteristics of a bolus developed based on a Polydimethylsiloxane (PDMS) monomer and curing agent (10:1); from the results, it was demonstrated that the bolus designed with a thickness of 0.5 and 1 cm meets the dosimetric characteristics necessary for a tissue-equivalent bolus suitable for radiotherapy. However, in our case, customized devices were developed based on dental wax-paraffin (50:50), with a thickness of 1 cm. The dental wax has a density of 1.02 g/cc and is distributed in thin rectangular sheets of dimensions 16.5 cm × 9.2 cm with a thickness of 1.5 mm. The average HU for the wax material in CT is −120, which is almost equal to that of chest wall tissue (average HU −100) [24], where the homogeneity and conformity indices were adequate. In addition, the benefits of the wax and paraffin with which the homogenizer was developed are characterized by their adequate tissue equivalence, dimensional and chemical stability, and moldability (131–158 °F). This allows its adaptation to the tumor surface regardless of the anatomical region [8]. Figure 5 shows the flow diagram of the methodological process for radiotherapy treatment, based on the design of homogenizing devices in our patients with non-melanoma skin cancer.

Despite the limited literature regarding homogenizers, studies conducted by Miéville et al. and Aras et al. [25,26] compared the dosimetric properties and clinical application between Superflat, Playdough material, and dental impression silicone in post-mastectomy patients with breast cancer. They found that customized homogenizers adapted better to the patient’s shape and offered a more precise dose distribution compared to prefabricated ones, regardless of their low cost and speed of use. On the other hand, in a study conducted by Verma et al. [27], they compared the use of virtual homogenizers with respect to prefabricated gel and wax homogenizers. The authors demonstrated that non-virtual homogenizers presented better consistency, which translates into a greater benefit in response to treatment, particularly in patients with irregular surface tumors. In this sense, it is important to highlight the importance of the dosimetric details constituted by the homogeneity and conformity index, which must comply with the recommended values, since these allow one to know the way in which the dose behaves or quantifies, as well as the distribution of radiation with respect to the size and geometry of the target volume, with indices as close to 1 as possible being suitable [28]. The above was a characteristic of the homogenizing devices designed for the cases reported in this work.

It should be noted that since these are customized devices, it is crucial to perform adaptive radiotherapy because sometimes the tumor shrinks, and a new customized homogenizer is necessary to achieve better coverage. This was the case with patient 3 in our study. Prior to treatment with the customized homogenizer, the skin toxicity was grade 1, whereas at the end of treatment, the skin toxicity was grade 2, according to the CTCAE [19]. Therefore, two customized 1 cm thick homogenizing devices allowed radiotherapy treatment to be carried out; however, despite successful treatment in the tumor regions, pulmonary and locoregional progression was evident, which caused his death.

According to the classification of non-melanoma skin tumors, the presented cases are high-risk patients [15,16]. This is due to the characteristics of lesions >20 mm in size located on the extremities and lesions >6 mm in the mask area. In addition, they have poorly defined borders and include histological subtypes such as basal cell carcinoma with an adenoid component, acantholytic carcinoma, and carcinomatous lymphangitis [15,16]. Although Mohs surgery would be the treatment of choice, this group of patients were not surgical candidates according to the multidisciplinary consultation at our hospital. Therefore, radiotherapy represented an excellent alternative to Mohs surgery with a reported control rate of 90–95% [29,30].

Although in our hospital, we have treated around 100 cases of various types of lymphomas, sarcoma, breast cancer, penile cancer, and skin cancer with radiotherapy and homogenizing devices for radiotherapy treatment, only three cases of skin cancer from a single center are presented, being the main limitation of this work.

## 4. Conclusions

The use of customized paraffin wax-based (50:50) homogenizing devices at our institution optimized the conformity and homogeneity indexes, achieving values that comply with the protocol for radiotherapy treatments in patients with irregular surface tumors.

In addition to the ease and cost of developing these devices, they reduced toxicity, facilitated planning, and improved dose distribution to the target volume, resulting in a better clinical response in the cases presented. Therefore, they represent an excellent alternative when Mohs surgery is impossible or the patient is simply not a candidate for surgical intervention.

## Figures and Tables

**Table 1:** Clinical characteristics of patients treated with radiotherapy and customized homogenizers.

Case	Histology	Location	Acute Toxicity	Chronic Toxicity	Dose	Clinical Response	Follow-Up	Conformity/ Homogeneity Index
1	Infiltrating solid basal cell carcinoma with adenoid component	Nasolabial fold	Radiation dermatitis grade 1	Grade 1 skin hypopigmentation	55 Gy/20 Fx	Complete locoregional response	2 years	0.4289/0.1274
	Infiltrating solid basal cell carcinoma with adenoid component	Right thigh	Radiation dermatitis grade 1	Grade 1 skin hypopigmentation	55 Gy/20 Fx	Complete locoregional response	2 years	1.4732/0.1366
2	Classic basal cell carcinoma	Inner corner of the left eye	Radiation dermatitis grade 1 Dry Eye Grade 1 Grade 1 eyelid retraction	Grade 1 skin hypopigmentation	55 Gy/20 Fx	Complete locoregional response	2 years	0.0988/0.0981
3	Acantholytic squamous cell carcinoma plus carcinomatous lymphangitis	Left shoulder	Radiation dermatitis grade 2	Grade 1 skin hypopigmentation	CTX: CPPD x 4 ciclos RTX: 66 Gy/33 Fx	Complete locoregional response Distant metastasis	1 year and four months	0.05569/0.10098 0.03150/0.10091

Gy: Grays; Fx: Fractions

## Data Availability

The original data presented in the study are included in the article, further inquiries can be directed to the corresponding author.
